# Baltimore Letter to Journal of American Medical Association

**Published:** 1891-08

**Authors:** 


### Baltimore Letter to Journal of American Medical Association.

(Extract.)

I send you the following compositions on George Washington, which are veritable curiosities in the way of preliminary accomplishment, showing as well as anything could the need of some standard at least of requirements in those who undertake the study of medicine. They were handed in by two candidates for matriculation in one of our medical colleges, one of whom had been a teacher of a public school. I am glad to say both were rejected.

I. "George Washington When a boy could not tel a boy could not tel a lie he cut one of his fathers Chury trees with his hatchet when Father called him and aske who cut the tree he said I cut it with my hatchet he became a man of zeal and industry was belove by all who knew him he fought many battels and by his cunning defeated Nepoleon he maried became the first president of the United states lived to a good old age.

Oct. 1st"

II. "jeneral Washington was born in Virginia he was a truthfull Boy a good Chresttian a noble man in war & in peace he was the firist President of the United States of Amarica he was and considered the fathar of our country By his bravery whe are a free and independent people to think and act as whe think Best he was honest in all his ways."

I give them as near literally as I can. Yours truly,

